# Tin Sulfide (SnS) Films Deposited by an Electric Field-Assisted Continuous Spray Pyrolysis Technique with Application as Counter Electrodes in Dye-Sensitized Solar Cells

**DOI:** 10.1021/acsomega.2c03454

**Published:** 2022-10-27

**Authors:** Tauheed Mohammad, Firoz Alam, Aditya Sadhanala, Hari M. Upadhyaya, Viresh Dutta

**Affiliations:** † Centre for Nanoscience and Engineering, Indian Institute of Science, Bangalore 560012, India; ‡ Department of Electronic and Electrical Engineering, University College London, London WC1E 6BT, U.K.; § Photovoltaic and Optoelectronic Device Group, Clarendon Laboratory, University of Oxford, Oxford OX1 2JD, U.K.; ∥ Cavendish Laboratory, University of Cambridge, JJ Thomson Avenue, Cambridge CB3 0HE, U.K.; ⊥ London Centre for Energy Engineering, School of Engineering, London South Bank University, London SE1 0AA, U.K.; # Department of Energy Science and Engineering, Indian Institute of Technology Delhi, New Delhi 110016, India

## Abstract

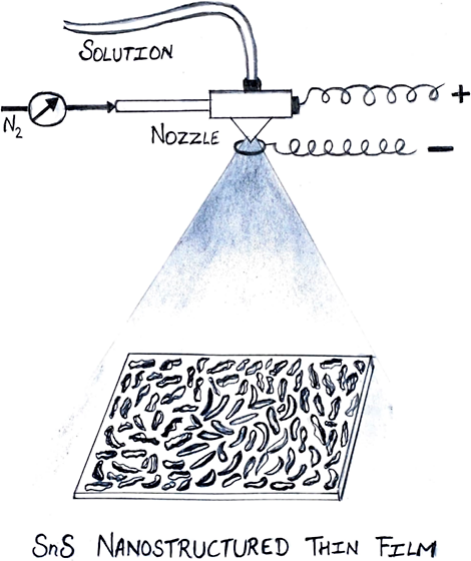

The deposition of tin sulfide (SnS) nanostructured films using a continuous spray pyrolysis technique is reported with an electric field present at the nozzle for influencing the atomization and the subsequent film deposition. In the absence of the electric field, the X-ray diffraction pattern shows the orthorhombic phase of SnS with a crystallographic preferred orientation along the (040) plane. The application of the electric field results in significant improvement in the morphology and a reduction in surface roughness (28 nm from 37 nm). The direct optical band gap of the films deposited with and without the electric field is estimated to be 1.5 and 1.7 eV, respectively. The photothermal deflection spectroscopy studies show a lower energetic disorder (no Urbach tail), which indicates an annealing effect in the SnS films deposited under the electric field. The improvement in the film properties is reflected in the expected improvement in the power conversion efficiency (PCE) of dye-sensitized solar cells fabricated using the SnS film as a counter electrode. An enhancement of PCE from 2.07% for the film deposited without the electric field to 2.89% for the film deposited with the electric field shows the role of the electric field in the fabrication of improved SnS films.

## 1 Introduction

Photovoltaic (PV) cells have emerged as a promising source of clean energy to meet current concerns of global climate change.^1^ As a third-generation solar cell, dye-sensitized solar cells (DSSCs) have received tremendous attention as one of the promising PV technologies, due to their low cost and simple fabrication, reasonable power conversion efficiencies, ease of building combination, and environmental friendliness.^2−4^ In order to improve the PV performance of DSSCs, modern efficient and stable dyes, outstanding light scattering ability, fast electron transfer, and a new photoanode architecture model with high specific surface area have been widely used for two decades.^5−7^ Most organic sensitizers include a pi-bridge as the passage for the electron transfer between the donor and the acceptor which is important for highly efficient DSSCs. Porphyrin-based sensitizers have an intense spectral response between 400–450 nm (Soret band) and 500–700 nm (Q-band), along with their good photo, chemical, and thermal stability, which has also attracted considerable attention from DSSC researchers.^8^ The great effort to improve the performance of DSSC devices based on conventional liquid electrolytes has been witnessed with recent achievement in a power conversion efficiency (PCE) of over 14%.^9^ To achieve practical long-term application of DSSCs, dye engineering is crucial to increase the PCE, while replacing the high-cost Pt counter electrode (CE) by producing inexpensive CEs to minimize the production cost. The CE of DSSCs should have high catalytic activity for the regeneration of redox couples and electrical conductivity for electron collection from the external circuit. Conventionally, Pt performs as a catalyst coated on the fluorine-doped tin oxide (FTO) substrate which acts as an electron collector and is used due to its high stability and catalytic activity.^10,11^ The high cost and limited reserves restrict Pt to be used as an appropriate CE catalyst, so many other low-cost Pt-free materials have been explored by the researchers such as carbon materials, multiple compounds, inorganic materials, polymers, and composites as CE catalysts.^12,13^ Another interesting material is tin sulfide (SnS) for CE applications whose *I*–*V* measurements with different metals (Au, Mo, and Ti) form an Ohmic contact and Schottky contact in Al–SnS–In and Ag–SnS–In systems, respectively.^14,15^ SnS is nontoxic, cheap, chemically stable, and naturally abundant, and the facile cost-effective synthesis makes it suitable for various PV applications.^16−18^ Also, it possesses optimal band gap (<2 eV), p-type nature, and high absorption coefficient (>10^4^ cm^–1^) with superior charge migration which is preferable for solar cells.^19,20^ It has been reported in the literature that the band gap of SnS is narrower than that of tin disulfide (SnS_2_) and the electron affinity and the ionization potential of SnS are also smaller than those of SnS_2_. Also, based on density functional theory calculations studies, SnS is expected to be highly useful as a photocathode for hydrogen production.^21^ To date, a range of established coating methods have been used to deposit SnS semiconducting thin films, namely, atomic layer deposition,^22^ chemical vapor deposition,^23^ thermal evaporation,^24^ sputtering,^25^ spray pyrolysis,^26^ electrochemical deposition,^27^ E-beam evaporation,^28^ and chemical bath deposition.^29^ However, the mentioned techniques are either expensive due to vacuum-based technology or suffer from slow deposition rate except spray deposition.

The spray technique is one of the most attractive deposition methods for low-cost and large-scale production with additional benefits of easy and accurate control over film fabrication. Recently, researcher’s center of attention is to develop particles with controlled shape and size since the optoelectronic and physicochemical properties are strongly modulated by it. Thus, the synthesis of metal sulfide thin films with a controlled nanostructured to tune the optoelectronic properties is important for commercial applications.^30^ Herein, we report a novel solution-processable shape–size-controlled synthesis of nanostructured SnS films on an FTO-coated glass substrate using a continuous spray pyrolysis (CoSP) technique with the applied electric field during the spray deposition. The properties of SnS films strongly depend on the growth methodology,^31^ and the effect of the electric field on the structure and morphology has been successfully illustrated using the deposited films as CEs in a tri-iodide/iodide (I_3_^–^/I^–^)-based DSSCs.

## 2 Experimental Details

Tin chloride (SnCl_4_·2H_2_O) procured from Alfa Aesar, thiourea, and methanol from Merck were used as precursors to make spray solution (tin chloride and thiourea both 0.05 M each in methanol). The CoSP technique is used to deposit SnS films with and without applied voltage on cleaned FTO substrates by spraying the well-mixed solution after ultrasonication into the furnace. These films are then used as CEs for DSSCs. The fabrication of DSSCs was carried out using [di-tetra butyl ammonium *cis*-bis (iso thio cyanato) bis (2, 2′-bipyridyl-4, 4′-dicarboxylato)] ruthenium(II) dye obtained from Sapala Organics Private Limited, India, which was infiltrated into the TiO_2_ mesoporous layer on the FTO substrates. TiO_2_ paste (18NRT, particle size ∼20 nm), obtained from Dyesol, Australia, was used to make the mesoporous layers. The layers are treated using titanium tetrachloride (TiCl_4_) obtained from Loba Chemie for obtaining a well-connected TiO_2_ layer. The electrolyte was prepared using IonLic DMPII from Solaronix, ethanol from Merck, and 4-*tert*-butylpyridine and acetonitrile from Sigma-Aldrich. All procured chemicals are utilized without any additional purification.

**Figure 1:**
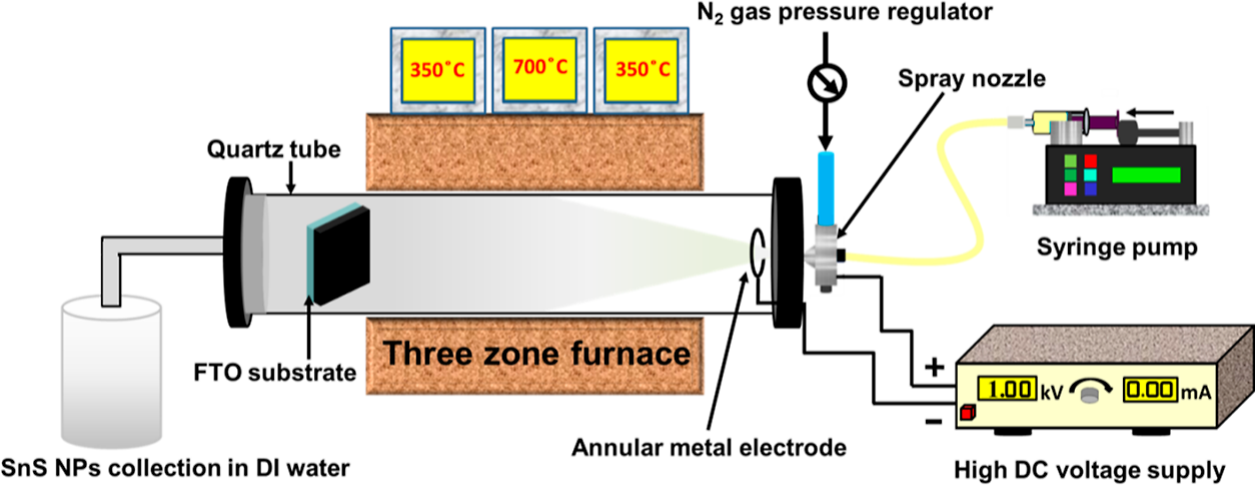
Graphic of the electric field-assisted CoSP technique.

The schematic of the electric field-assisted CoSP technique is depicted in Figure 1. The spray solution (through the connecting Teflon tubes) from a programmable infusion pump and the carrier gas (nitrogen) are sprayed into the three-zone furnace using a 1/4J series spray nozzle (Spraying System Co., USA). The temperatures in three zones are maintained at 350, 700, and 350 °C. The cleaned FTO substrates were placed in the third zone to obtain SnS-deposited films, while rest of the nanoparticles formed were collected in the collection chambers. SnS films are also deposited using smaller size nanoparticles created using an additional electric pressure during atomization which can be achieved by associating 1000V DC power supply to the nozzle, and the circular metallic electrode is kept at ∼4 mm inside the furnace. Hence, droplet size further reduced with the application of applied DC voltage due to the Coulombic fission process.^32^ The SnS films thus created will help in understanding the role of the electric field in the self-assembly of the nanoparticles for the film formation on the substrate.

## 3 Device Fabrication

Glass substrates coated with FTO were subsequently cleaned with ultrasonication at 50 °C for 30 min each in Labolene solution, deionized water, acetone, and isopropyl alcohol. Nitrogen was used to dry the cleaned substrate, and then, it was preheated at 100 °C for 60 min before the final use. The TiO_2_ photoanode and DSSC devices were fabricated according to a previous report.^33^ The excessively aggregated dye molecules in the photoanode were removed by ethanol rinsing and flushed with N_2_. Finally, the dye-loaded photoanode was sandwiched with different CEs. The SnS film spray-coated without using the DC voltage (0 V) is named SnS-0V, and the film fabricated with an applied DC voltage of 1000 V is named SnS-1000V. The liquid electrolyte of I^–^/I_3_^–^ was filled in between the two electrodes. All device steps reported were conducted and measured under ambient laboratory conditions in air with 0.25 cm^2^ active area of devices.

## 4 Characterization Techniques

A Rigaku Ultima IV X-ray diffractometer equipped with a Cu K_α_ X-ray tube (λ = 1.54056 Å) was used for analysis of SnS thin films. Surface morphologies were obtained using a Hitachi S-4300 scanning electron microscopy (SEM) system, and the tapping mode of atomic force microscopy (AFM) was used to examine surface roughness. A PerkinElmer Lambda (1050) UV–vis–NIR spectrophotometer was used for absorbance spectral measurements, and a Dektak-XT stylus surface profiler was used for thickness measurements. Photothermal deflection spectroscopy (PDS) is used to measure the optical absorption of SnS films near the band edge down to 10^–5^ and described by Sadhanala et al.^34^ The current-density (*J–V*) measurements of both platinum (Pt) and SnS CE-based DSSCs were conducted with an Oriel class 3A Newport solar simulator under AM1.5 simulated solar illumination (100 mW cm^–2^).

## 5 Results and Discussion

**Figure 2:**
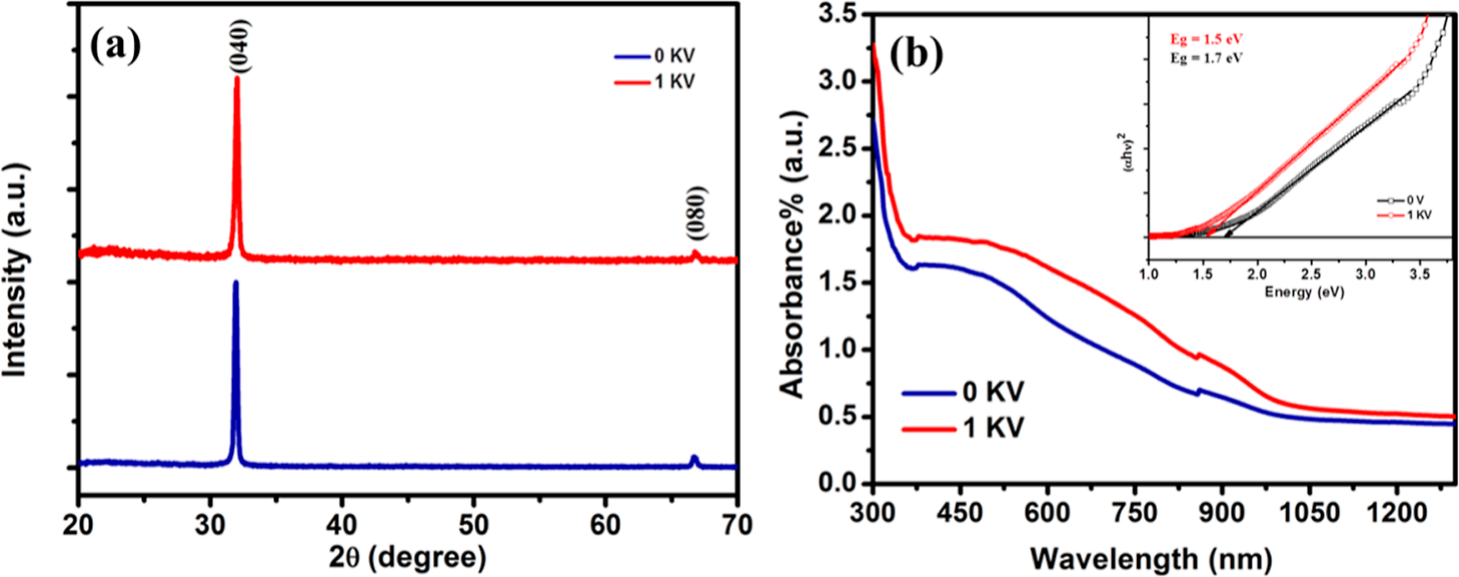
(a) Normalized XRD patterns. (b) UV–vis absorbance spectra with Tauc’s plot (inset) of SnS films prepared by the CoSP technique with and without the electric field.

SnS films deposited by the CoSP technique with a thickness of ∼500 nm possess excellent adhesion to the substrate. Figure 2a shows the normalized X-ray diffraction pattern of the SnS films deposited with and without the electric filed. The dominant sharp and narrow peak centered at 31.9° is assigned to the (040) orientation of the orthorhombic phase of SnS [JCPDS no. 39-0354, lattice parameters *a* = 4.329 Å, *b* = 11.192 Å, and *c* = 3.984 Å, space group Pbnm (62)]. It is suggested that the deposited material is highly crystalline and has a preferred crystallographic orientation in the film along the (040) direction. The spray-deposited SnS nanostructured film consisting a single phase of SnS has already been observed before.^35^

The optical characteristics of CoSP-deposited SnS thin films with and without the electric field are illustrated by absorption spectra, as shown in Figure 2 b. It is noticed that the absorption increases noticeably for the SnS film fabricated using the electric field. This result is analogized with the surface morphological improvement of SnS films deposited using electric field-assisted CoSP, as described in the SEM discussion. The energy band gap values of SnS thin films calculated from the Tauc’s plot are presented in the Figure 2 b inset. The energy band gap is found to be 1.7 and 1.4 eV for the SnS thin film prepared by CoSP at applied 0 and 1000 V, respectively. The decrease in the value of the band gap for the SnS film fabricated using the applied electric field is attributed to charging and variation of droplet size to ultrafine drizzle during the spray deposition.

**Figure 3:**
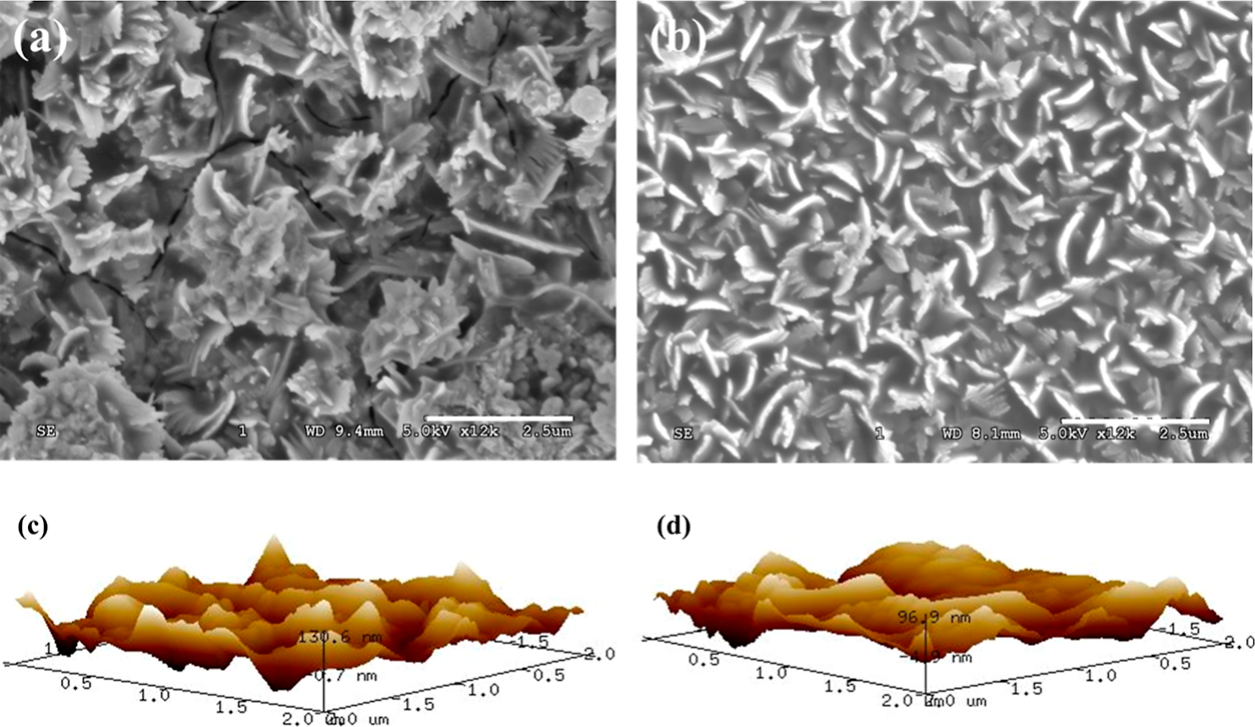
SEM and AFM images of spray-deposited SnS nanostructured films on FTO-coated glass at applied voltage during the deposition at 0 V (a,c) and 1 kV (b,d) respectively.

The application of an electric field with running solution plays a notable role in electrostatic atomization of liquid during spray deposition without a tiny orifice and high pressure of carrier gas.^36^ Since Coulombic repulsion overcomes the binding force of droplets due to the net charge density available on the surface of the droplet, disruption of unstable droplets occurs, and hence, atomization rate increases, termed Coulombic fission.^37^ In this experiment, solution-processable SnS thin films were ab initio fabricated where reacting atomic species resulted in individual nucleation centers on a heated substrate, encouraging the growth of randomly distributed nanoflakes, and the solvent evaporated simultaneously to bestow interesting properties to thin-film materials. Figure 3a,b shows SEM micrographs of CoSP-deposited SnS thin films without and with an applied electric field. It is clearly seen from SEM images that there is an agglomeration and there are cracks in the films (Figure 3a) deposited without the electric field due to larger droplet size, but a continuous and homogeneously distributed nanoflake film without any cracks (Figure 3b) and which is well adherent to the substrate is obtained with the applied electric field. These randomly distributed nanoflakes are providing large surface area for effectively reflecting the unabsorbed light back to the cell which increases the optical path and hence probability of photons to get absorbed by dye molecules.^38^ Also, nanoflakes are well interconnected for effective charge mobility to complete the device circuit, as CEs fetch electrons from the outer circuit and transfer them into the cell which is proved by enhanced PV performance of DSSC. Thus, the electric field-assisted CoSP technique successfully established nanostructured selective deposition of SnS thin films explained by SEM studies. Improved surface morphology and roughness reduction of SnS thin films due to the applied electric field is also confirmed by SEM and AFM results, respectively. The tapping mode AFM images of CoSP-deposited SnS thin films are shown in Figure 3. The SnS film without the application of DC voltage produces irregular surface morphology (Figure 3 c) with a root mean square roughness (*R*_rms_) of 37 nm, which is ascribed to generation of uneven larger droplets during the spray, and *R*_rms_ roughness of the SnS film deposited with the electric field reduced to 22.6 nm (Figure 3 d), which results in better interface connection and improved charge collection efficiency.

**Figure 4:**
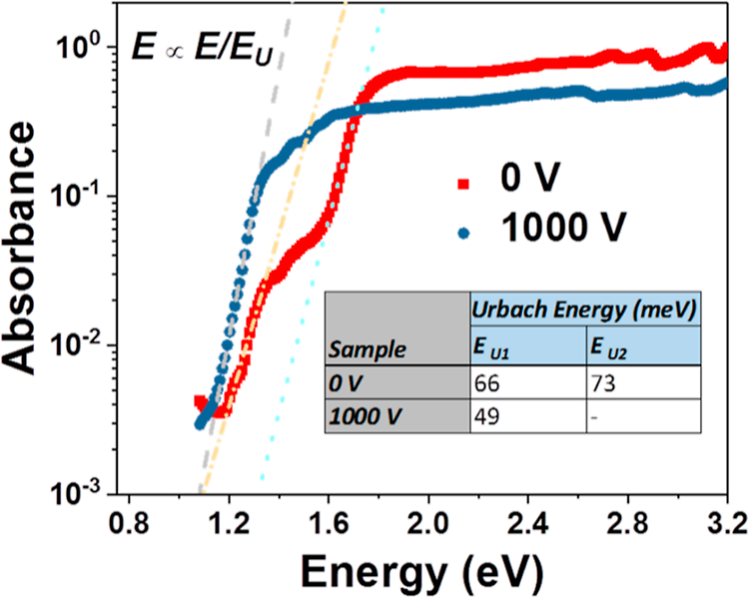
PDS of SnS layers prepared by the CoSP technique without and with the electric field.

To understand the nature and quality of the SnS thin films formed, we performed PDS measurements. PDS is one of the most sensitive absorption measurement techniques capable of measuring absorption with 4–5 orders of magnitude dynamic range. This would enable us to look at the sub-band gap states present in our samples, and we can estimate the energetic disorder in our samples by fitting the exponentially falling absorption tail. This energetic disorder is expressed as Urbach energy “*E*_U_” and is estimated from the absorption *A* ∝ exp(*E*/*E*_U_).^34^ Urbach energy is an implicit way of assessing the quality of a thin film.^34^Figure 4 shows PDS spectra of CoSP-deposited SnS thin films without and with the applied electric field. For our SnS thin film with no electric field (0 V), we observe two band edges with the highly absorbing band tail demonstrating an Urbach energy of 66 meV and the less absorbing band tail demonstrating an Urbach energy of 73 meV. However, the SnS thin film deposited under a 1000 V electric field demonstrates a single band edge with an Urbach energy of 49 meV. This gives us two inferences—first being the complete conversion of SnS in the case of electric field-assisted SnS deposition demonstrating single band edge as compared to a possible two-phased SnS thin film obtained when not applying any electric field, and further investigations regarding the origins of this are currently underway and are part of a separate study. Second important inference is the low energetic disorder obtained in SnS thin-film samples prepared under the electric field. These inferences present the positive advantage of using the electric field while depositing high-quality SnS thin films.

**Figure 5:**
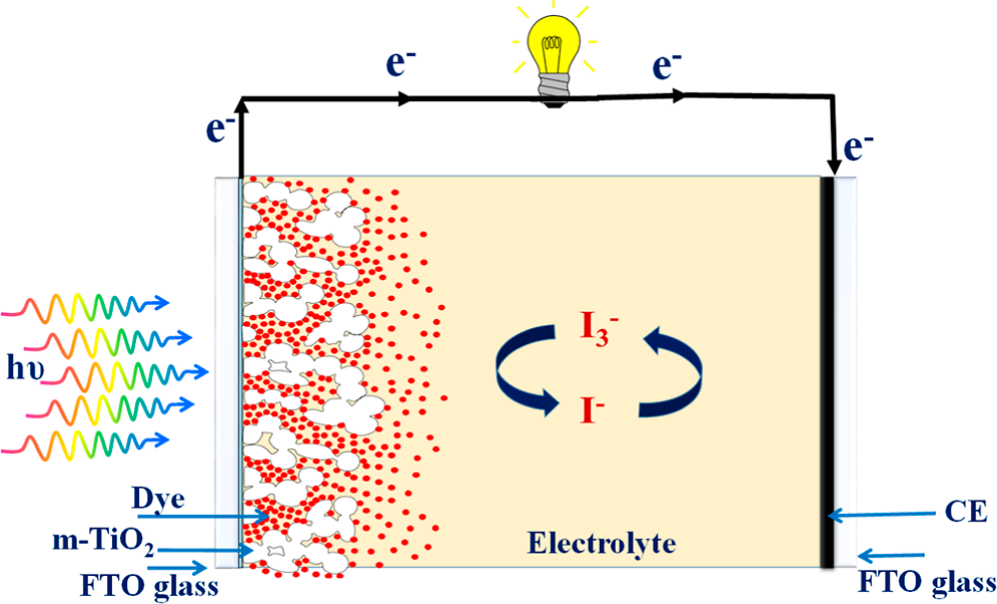
Schematic diagram of the DSSC device structure.

**Figure 6:**
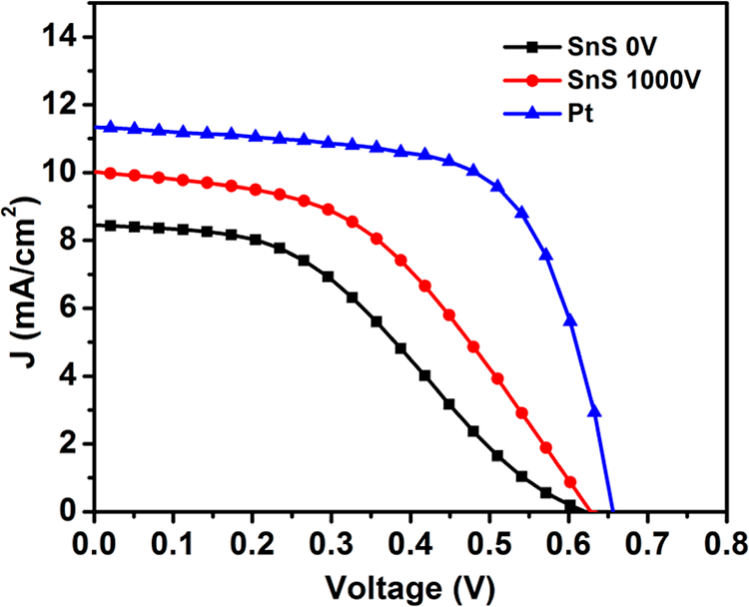
Current density–voltage (*J*–*V*) characteristics of the DSSCs using these SnS films and Pt as CEs.

The best performing cathode should be cheap and porous have catalytic activity, high surface area, and good adhesivity with FTO for efficient entry of electrons into the cell, and reduction occurs.^38^ The oxidized redox couple is reduced by accepting electrons at the CE, and the oxidized dye molecules are reduced by accumulating electrons via the electrolyte in DSSCs.^39^ The PV performance of several DSSCs with different CEs is summarized in Table 1, and the scheme of a DSCC is shown in Figure 5. The current density–voltage (*J*–*V*) characteristics of deposited SnS films as CEs with and without the electric field are shown in Figure 6. The PV performance parameters compared with reference conventional DSSCs with Pt as CEs are summarized in Table 2.

**Table 1:** PV Efficiency of Various Materials Used as the CE in the Fabrication of DSSCs

CE material	synthesis technique	efficiency η (%)	reference
WS_2_	chemical method	7.73	(40)
PbS	chemical method	6.49	(41)
SnS nanowires	hydrothermal	5.00	(42)
MoS_2_	electrodeposition	4.84	(43)
CZTS	solution phase	3.62	(44)
SnS	electric field-assisted CoSP	2.89	present work
SnS_2_	solvothermal	2.82	(45)
Co-SnS_2_	hydrothermal	2.56	(46)
CdS	chemical method	2.45	(41)
CoS	hydrothermal	2.40	(47)
NiS	hydrothermal	2.05	(47)
SnS	spray pyrolysis	2.00	(35)

**Table 2:** PV Performance Parameters of SnS-0V, SnS-1000V, and Pt CE-Based DSSCs

device	*V*_oc_ (V)	*J*_sc_ (mA/cm^2^)	FF (%)	η (%)
SnS 0V	0.622	8.42	39	2.07
SnS 1000V	0.627	10.00	46	2.89
Pt	0.656	11.34	66	4.89

The device fabricated with the SnS CE (deposited at 0 V) exhibited an open-circuit voltage (*V*_oc_) of 0.62 V, short-circuit current (*J*_sc_) of 8.42 mAcm^–2^, fill factor (FF) of 39.4%, and PCE of 2.07%. Device performance was dramatically enhanced by applying the electric field during spray deposition which is attributed to a significant increase in *J*_sc_ to 10 mAcm^–2^ and improved FF to 46%. As a result, the PCE was increased to 2.89% due to improved morphology and reduced roughness which facilitates better interface and improved overall charge collection efficiency. The applied electric field has a great influence on shape, size, surface area, morphology,^36^ and the catalytic property of the SnS CE. The developed nanoflakes provide larger surface areas which will produce more catalytic active sites and support the improvement of electrocatalytic activity of the SnS CE. The overall performance of the devices prepared under similar conditions using the as-deposited and electric field-assisted SnS nanostructured CEs is lower than that of the standard platinum (Pt) CE (4.89%).

## 6 Conclusions

SnS films have been deposited on cleaned FTO substrates with and without the electric field using the CoSP technique, and their application as a CE in DSSCs has been successfully demonstrated. The sharp (040) reflection peak is centered around 31.9° indexed to the orthorhombic phase of SnS with a crystallographic preferred orientation along this direction. The band gap values were observed to be 1.7 and 1.4 eV for the SnS thin film without and with the applied electric field, respectively. The deposited SnS film with the applied electric field revealed the randomly distributed nanoflakes without any cracks in SEM images and reduced roughness in AFM topological images. The improvement in the surface roughness, morphology, and complete conversion of SnS with low energetic disorder due to the applied electric field results in the enhancement of PCE of devices. Hence, the solution-processable shape–size-controlled synthesis of nanostructured SnS films using the CoSP technique with the application of the electric field during the spray deposition is established.
